# Procedural and Therapy Peripheral Intravenous Catheters: A Framework for Safety, Quality and Patient‐Centred Care

**DOI:** 10.1111/jan.70347

**Published:** 2025-11-03

**Authors:** Bertrand Drugeon, Claire M. Rickard, Nathan Peters, Vineet Chopra, Olivier Mimoz

**Affiliations:** ^1^ Service des Urgences Adultes—SAS 86—SAMU, CHU de Poitiers Poitiers France; ^2^ INSERM, PHAR2, U1070, Pharmacologie Des Agents Anti‐Infectieux Poitiers France; ^3^ Alliance for Vascular Access Teaching and Research Griffith University Brisbane Queensland Australia; ^4^ School of Nursing, Midwifery and Social Work and Centre for Clinical Research University of Queensland Brisbane Queensland Australia; ^5^ Faculté de Médecine et de Pharmacie Université de Poitiers Poitiers France; ^6^ Herston Infectious Diseases Institute, Metro North Health Brisbane Queensland Australia; ^7^ Department of Anaesthesia and Perioperative Medicine The Royal Brisbane and Women's Hospital Brisbane Australia; ^8^ Faculty of Medicine The University of Queensland Brisbane Queensland Australia; ^9^ The Hospital Medicine Safety Consortium Coordinating Center Ann Arbor Michigan USA; ^10^ Department of Medicine University of Colorado Anschutz Medical Campus Aurora Colorado USA

**Keywords:** catheter‐related complications, occlusive dressing, patient‐centred care, peripheral intravenous catheter, skin antisepsis, vascular access management

## Abstract

**Aim:**

To introduce a novel conceptual framework that differentiates peripheral intravenous catheters according to their dwell time and therapeutic purpose, in order to improve the suitability of material choice, safety and cost‐effectiveness.

**Design:**

Concept based on clinical guidelines, expert consensus and recent peer‐reviewed evidence.

**Data Sources:**

A literature search was conducted in PubMed on November 25, 2024, using defined keywords related to peripheral intravenous catheters, device complications and duration of use. This search was supplemented by manual screening of references from relevant articles.

**Methods:**

The analysis followed the SANRA quality criteria for narrative reviews. Evidence and recommendations from clinical guidelines, randomised trials and qualitative studies were synthesised using the Australian Clinical Care Standards to structure the proposed classification into ‘procedural’ and ‘therapy’ catheters.

**Results:**

Procedural catheters are used for less than 24 h, typically during procedures or short treatments, and are mainly linked to immediate risks like insertion failure and local trauma. Therapy catheters, defined as devices used beyond 24 h or expected to remain while the patient sleeps, carry cumulative risks, including delayed complications such as phlebitis, occlusion and infection. The framework supports more nuanced decisions on device choice, insertion site and maintenance.

**Conclusion:**

This framework introduces a practical differentiation between short‐ and longer‐term peripheral venous catheters, addressing a major oversight in existing guidelines and supporting context‐sensitive vascular access decisions.

**Implications for the Profession and/or Patient Care:**

Tailoring catheter management to expected dwell time may reduce complications and costs, enhance workflow, and improve patient comfort.

**Impact:**

By addressing the lack of temporal distinction in current practice, this framework offers a simple yet transformative tool applicable across care settings, with the potential to improve patient outcomes, resource utilisation and costs.

**Patient or Public Contribution:**

This project is a concept analyses; no patient or public contribution was necessary.

**Abbreviations**
ACCS: Australian Clinical Care Standards
aRR: adjusted risk ratio
AU$: Australian dollar
CHG: chlorhexidine gluconate
CRBSI: catheter‐related bloodstream infection
CVC: central venous catheter
DIVA: difficult intravenous access
ED: Emergency Department
IPA: isopropyl alcohol
PIVC: peripheral intravenous catheter
PVI: povidone‐iodine
RCT: randomised controlled trial
RR: relative risk

**Summary**

- This paper introduces a novel *procedural/therapy PIVC* framework based on expected dwell time and therapeutic purpose.
- The framework enables more appropriate decision‐making regarding device choice, insertion strategy, and maintenance protocols.
- Adopting the *procedural/therapy PIVC* model may improve patient outcomes, optimise resource use, and facilitate guideline implementation.

## 1 Introduction

Peripheral intravenous catheter (PIVC) is the most commonly used medical device in hospitals for administering medications, blood products, contrast agents for radiological investigations, fluids and for blood sampling (Alexandrou et al. 2018; Hadaway 2012). However, its insertion is not without risk, with first‐attempt failure rates reaching 40% and post‐complications occurring in 50% of cases (Witting 2012; Helm et al. 2015; Paterson et al. 2022; Sebbane et al. 2013; Lapostolle et al. 2007).

Complications are frequent and multifaceted. Early complications include insertion failure, multiple puncture attempts, infiltration, and local trauma, leading to patient pain, anxiety and treatment delays (van Loon et al. 2016; Schults et al. 2022). During use, catheters are prone to mechanical issues such as occlusion, dislodgement, and accidental removal, as well as local inflammatory reactions such as phlebitis (Marsh et al. 2020). Infectious complications are less common but more clinically significant, ranging from local site to bloodstream infection, with associated morbidity, prolonged hospitalisation, and increased costs (Marsh et al. 2024).

Beyond these recognised risks, many PIVCs are placed unnecessarily, either as a precautionary measure ‘just in case’ or for anticipated treatments that are ultimately not required (Xu et al. 2023; Hawkins et al. 2018; Egerton‐Warburton et al. 2019). Such unnecessary catheters expose patients to the harms of insertion and dwell without delivering any clinical benefit, representing both a patient safety issue and a waste of healthcare resources (Morgan et al. 2022). In practice, even when clinically indicated at insertion, PIVCs are often left in situ once their purpose is fulfilled, resulting in idle devices at risk of avoidable complications.

## 2 Background

PIVCs are primarily inserted and managed by nurses, who play a central role in ensuring safe, evidence‐based vascular access care. Multiple guidelines and safety standards have been developed to improve PIVC management. Collectively, they emphasise that PIVCs should only be inserted when clinically indicated, with daily reassessment of ongoing need to avoid unnecessary devices. Insertion should follow strict aseptic technique, including appropriate skin antisepsis and site preparation, performed by trained staff to maximise first‐attempt success and minimise complications. Device choice and insertion site selection should be tailored to the prescribed therapy and anticipated duration, with preference for peripheral rather than central devices whenever safe and feasible. Once in place, PIVCs require securement with sterile dressings, routine inspection, and flushing protocols to maintain patency and detect early signs of phlebitis, infiltration or infection. Finally, prompt removal is recommended as soon as the device is no longer needed or if complications arise (Nickel et al. 2024; World Health Organization 2024; Moureau and Chopra 2016; Australian Commission Safety Quality Health Care 2021).

Several studies have documented persistent gaps between guidelines content and bedside practices, driven by factors such as high workload, limited resources, insufficient training, and the widespread perception that PIVC insertion is a routine, low‐risk procedure rather than an invasive intervention requiring strict adherence to best practice (Alexandrou et al. 2018; Xu et al. 2023). In addition, the generic nature of most guidelines makes them difficult to operationalise across the wide range of clinical settings where PIVCs are used. These devices serve multiple purposes, from rapid access in emergencies, to contrast administration in radiology, to short‐term use in procedural suites, and even prolonged therapy in hospitalised patients requiring antibiotics, hydration or analgesia. Each of these contexts presents distinct workflow pressures, patient populations and resource constraints. As a result, recommendations designed to be broadly applicable often fail to accommodate the realities of clinical practice, limiting their uptake and consistent implementation (Xu et al. 2023). Taken together, these limitations suggest that while current approaches to PIVC management are evidence‐based, they remain overly generic and insufficiently adapted to the heterogeneous nature of PIVC use. Addressing this gap requires approaches that better reflect the diverse clinical scenarios in which PIVCs are used.

## 3 Data Sources

### 3.1 Design

A narrative review was chosen because it enables the integration of diverse types of evidence, including clinical guidelines, expert consensus, position papers and practice‐based recommendations. Unlike systematic reviews, which are constrained by rigid eligibility criteria, narrative reviews offer flexibility to synthesise heterogeneous data and to address underexplored questions in clinical practice. In the present study, this approach was particularly appropriate to support a concept analysis aiming to propose a new framework for PIVCs management.

The review was conducted and reported in accordance with the Scale for the Quality Assessment of Narrative Review Articles (SANRA), which recommends: (i) a clear justification of the relevance of the topic, (ii) explicit objectives, (iii) a transparent description of the literature search, (iv) adequate referencing, (v) scientific reasoning and critical discussion and (vi) a coherent presentation of evidence and conclusions (Baethge et al. 2019).

### 3.2 Search Method

A comprehensive literature search was conducted in PubMed on November 25th, 2024, using predefined keywords and MeSH terms related to PIVCs, dwell time, device complications and vascular access management. To enhance comprehensiveness and minimise the risk of missing relevant studies, we also applied a snowballing strategy: the reference lists of all included articles, as well as those of key international and national guidelines, were manually screened to identify additional relevant publications. This iterative process (snowball effect) allowed us to capture sources that may not have been indexed in PubMed or missed by the initial search terms, such as international guidelines, consensus statements and position papers from professional societies. In addition, the authors' reference collections were consulted to ensure inclusion of seminal and highly relevant studies in the field. The full search strategy and eligibility criteria are reported in Data S1.

### 3.3 Eligibility Criteria

Publications were included if they focused on PIVC insertion, maintenance or removal, particularly in relation to expected dwell time, complication prevention and device management. Eligible sources comprised:

- Clinical studies
- Clinical guidelines and consensus statements,
- Expert opinion papers,
- Narrative, scoping or systematic reviews.

We restricted inclusion to peer‐reviewed articles published in English or French. We excluded studies that focused exclusively on other catheter types (central venous catheters, arterial catheters, dialysis catheters, umbilical catheters, intraosseous access, midline catheters, non‐intravascular catheters), non‐clinical studies (e.g., laboratory or animal data), and publications lacking sufficient methodological details.

### 3.4 Data Extraction, Concept Analysis and Synthesis

Screening and data extraction were performed by the first author using predefined eligibility criteria. The PubMed search yielded 1304 records. After title and abstract screening, and incorporating additional relevant publications identified through snowballing and the authors' reference collections, a total of 75 articles were retained for inclusion in the synthesis. Extracted information included study type, focus (e.g., insertion technique, complication prevention, device choice, insertion site), and reported implications for PIVC management.

Findings were narratively synthesised, driven by the 10 safety and quality standards of the Australian Commission on Safety and Quality in Health Care (Table 1). This structured approach allowed mapping current evidence, highlighting convergence and divergence between guidelines, and identifying gaps in knowledge. The synthesis then informed a concept analysis aiming to generate a pragmatic framework for PIVC care.

**TABLE 1:** Summary of ACCS.

Number	ACCS	Description
1	Assess intravenous access needs	Ensure that a PIVC is necessary and consider alternative routes of administration before insertion
2	Inform and partner with patients	Provide clear information on the indication, procedure, and potential risks, and obtain the patient's consent
3	Ensure competency	PIVC insertion and management should only be performed by trained and competent healthcare professionals
4	Choose the right insertion site and PIVC	Choose the most suitable insertion site and catheter type based on clinical needs and patient preferences
5	Maximise first insertion success	Increase the likelihood of successful PIVC insertion on the first attempt, escalating to a more experienced clinician if needed
6	Insert and Secure	Follow standard precautions, including aseptic technique, and use appropriate dressing to secure the catheter
7	Document decisions and care	Ensure accurate documentation of PIVC insertion, maintenance, and removal for patient safety and continuity of care
8	Routine use: inspect, access and flush	Inspect the insertion site at least every 8 h and perform regular flushing to maintain patency
9	Review ongoing need	Reassess the necessity of the PIVC each day and remove it as soon as it is no longer required
10	Remove safely and replace if needed	Remove the PIVC at the first sign of malfunction or complications, and replace it only if ongoing intravenous access is necessary

### 3.5 Findings

The literature review identified several recurring themes in the management of PIVCs, which were organised according to the Australian Clinical Care Standards (ACCS).

#### 3.5.1 Insertion‐Related Issues (ACCS 1, 5)

High first‐attempt failure rates (ranging from 30% to 50%) (Witting 2012; Paterson et al. 2022; Sebbane et al. 2013; van Loon et al. 2016; Sweeny et al. 2022; Yalçınlı et al. 2019) and unnecessary ‘just‐in‐case’ insertions (Hawkins et al. 2018; Morgan et al. 2022; Mailhe et al. 2020; Noel et al. 2022) were repeatedly reported across emergency and ward settings. These practices contribute to patient discomfort, increased workload and resource waste (Drugeon et al. 2023; Lim et al. 2019; Maunoury et al. 2022; González López et al. 2014). Qualitative studies also described how time pressure and uncertainty about treatment plans drive precautionary insertions (Noel et al. 2022; Carr et al. 2016).

#### 3.5.2 Complication Prevention (ACCS 2, 3, 6, 8)

Guidelines (Nickel et al. 2024; World Health Organization 2024; Moureau and Chopra 2016; Australian Commission Safety Quality Health Care 2021) and studies (Rickard et al. 2023, 2018; Guenezan et al. 2021) consistently emphasised delayed complications such as phlebitis, infiltration and infection, while early issues like insertion failure or local trauma received less attention (van Loon et al. 2016; van Loon, Puijn, et al. 2018). This imbalance suggests that the immediate risks of short‐term catheters may be underestimated in current standards of care.

#### 3.5.3 Device Selection and Insertion Site (ACCS 4, 6)

Evidence linked device choice and insertion site to expected dwell time. Distal sites such as the hand and wrist were associated with easier insertion and higher success rates in emergency and short‐stay patients (van Loon et al. 2016, 2019; Sweeny et al. 2022), whereas forearm veins and integrated devices were linked to reduced phlebitis and longer dwell times in hospitalised or therapy‐dependent patients (González López et al. 2014; Rickard et al. 2023).

#### 3.5.4 Documentation and Catheter Removal (ACCS 7, 9, 10)

Litterature highlighted frequent lapses in documentation (Alexandrou et al. 2018; Aghdassi et al. 2019; Berger et al. 2022; Høvik et al. 2020), reassessment, and timely removal of PIVCs (Ray‐Barruel et al. 2023, 2018; Ray‐Barruel 2022). Idle or unused catheters often remained in situ beyond their intended purpose, exposing patients to avoidable complications (Alexandrou et al. 2015).

#### 3.5.5 Overall Synthesis

Across studies, a consistent message emerged: although dwell time and duration of therapy are repeatedly recognised as key determinants of the risk of complication, guidelines and research rarely distinguish between the different clinical purposes of PIVCs. This conceptual gap, between evidence acknowledging dwell time and the absence of explicit differentiation in practice, provided the rationale for developing the new PIVC framework proposed in this paper.

## 4 Overview of the Concept

### 4.1 Catheter Safety and Quality—A Brief Procedure or A Longer Journey?

To address this gap, we developed a pragmatic framework based on the findings of the literature review, which highlighted the lack of explicit differentiation between short‐term and longer‐term PIVCs. We propose categorising PIVCs into ‘procedural’ PIVCs, intended for short‐term procedural use (removed on the day of insertion or < 24 h), and ‘therapy’ PIVCs, designed for prolonged therapies (requiring the patient to sleep for at least one night with the PIVC). For example, a *procedural PIVC* placed for a CT scan or brief sedation carries immediate risks such as insertion failure or local trauma, whereas a *therapy PIVC* used for continuous antibiotics or pain management is more prone to delayed complications like phlebitis or infection.

The procedural/therapy distinction is easily understood by both patients and busy clinicians and guided by a crucial and straightforward question for the inserter: Is the catheter being inserted for a short procedure or for ongoing therapy? Around half of all PIVCs placed in hospitals are (or could be) *procedural PIVCs*, with the bulk being inserted in emergency, radiology, procedural suites and anaesthetic departments. This question is crucial as it should guide decisions on catheter type, insertion site, fixation and maintenance. Considering the intended duration of use prevents costly errors, such as using advanced devices for short‐term needs or neglecting safety measures for prolonged use. By encouraging deliberate, evidence‐based decisions, this framework aligns care with patient needs and optimises resources and patient comfort (Schults et al. 2022; Larsen et al. 2017). By clearly defining the indications and strategies for each PIVC type, the framework simplifies the application of international guidelines, addressing criticisms of their generic nature and implementation challenges. Its clarity reduces ambiguities in decision‐making, discourages inappropriate practices, and improves adherence to guidelines, ultimately enhancing patient safety and care efficiency.

This proposed framework introduces and critically evaluates this innovative *procedural/therapy PIVC* framework, aiming to optimise catheter management, reduce preventable complications, and improve patient outcomes, experiences and healthcare efficiency.

This framework is not intended to serve as clinical practice guidelines. Instead, it represents a conceptual proposal informed by the synthesis of existing evidence and by reasoning grounded in the timing of PIVC‐related complications. The *procedural/therapy* distinction should therefore be interpreted as an exploratory taxonomy designed to support reflection, guide research and complement, not replace, existing international recommendations. Its purpose is to provide a pragmatic lens through which clinicians and researchers may better understand the heterogeneity of PIVC use and related risks, while recognising that further empirical validation is needed before formal implementation.

### 4.2 ACCS 1: Assess Intravenous Access Needs

Before initiating intravenous therapy, clinicians should assess whether an alternative route of administration could be appropriate, such as oral or, where clinically justified, intramuscular, subcutaneous or rectal routes. The decision should be based on the patient's condition, the nature of the treatment, and the expected duration of therapy.

However, identifying the correct clinical indication for PIVC insertion is essential for appropriate patient care, avoiding unnecessary or ‘just in case’ PIVC insertions (Morgan et al. 2022; Sneyers et al. 2025; Evison et al. 2021). The decision should be based on the treatment type, chemical properties and expected duration. A clear initial indication ensures the PIVC is necessary and clinically justified, with oral or other routes of treatment or no treatment strongly considered. Differentiating between *procedural and therapy PIVCs* at insertion allows clinicians to select the most suitable device and management strategy, but in situations of clinical uncertainty, this distinction may be more difficult to establish at the time of insertion. For *procedural PIVCs*, this assessment can be particularly challenging due to diagnostic and therapeutic uncertainties in acute care settings. The urgency to establish intravenous access in patients with evolving conditions or undetermined treatment plans often leads to precautionary insertions, increasing the risk of unnecessary catheter use (Noel et al. 2022).

Evidence suggests that *procedural PIVCs* are best used for short‐term needs and may safely be removed once their intended purpose is fulfilled. This category typically includes PIVCs inserted in emergency settings for short diagnostic or procedural purposes, or perioperative catheters removed within the same day once oral intake is resumed.

*Therapy PIVCs* are intended for therapies overnight or longer and suitable for overnight hospital stays with ongoing treatments, continuous infusions or repeated therapies. Selecting devices designed for long‐term use ensures consistent access and limits the need for repeated insertions.

*In cases of uncertainty*, such as in EDs, it can be challenging to choose between a *procedural or therapy PIVC*. PIVCs should only be inserted when necessary and guided by clear clinical indications to avoid unnecessary procedures and devices; simple venipuncture should be prioritised over PIVC insertion wherever possible (Hawkins et al. 2018; Egerton‐Warburton et al. 2019; Lim et al. 2020). A *therapy PIVC* would be justified for patients clearly needing IV therapy for more than a day such as trauma patients, sepsis, and those at risk of clinical deterioration, such as instability, abnormal ECG or significant blood loss (Egerton‐Warburton et al. 2019).

### 4.3 ACCS 2: Inform and Partner With the Patient

Guidelines emphasise the importance of obtaining informed consent before inserting a PIVC (Nickel et al. 2024; Australian Commission Safety Quality Health Care 2021). This involves a shared decision between the inserter, prescriber, and patient to ensure the clinical need is clear, the insertion site is appropriate, and the patient understands the purpose and duration and how to keep themselves safe. The aim is to provide the patient with the best strategy for which he/she agrees. Discussing previous PIVC experiences, such as insertion difficulties, sites used, and concerns, allows clinicians to adapt their approach, reducing anxiety and improving first‐attempt success. For patients with difficult intravenous access (DIVA), discussing prior challenges and using techniques like advanced inserters and ultrasound guidance reduces anxiety and improves trust.

Patient‐reported studies frequently identify two main complaints: multiple insertion attempts and discomfort affecting sleep (Schults et al. 2022; Larsen et al. 2017). While guidelines recommend avoiding joint placements to reduce post‐insertion failure, these recommendations are largely based on studies of longer‐term catheters. In practice, prioritising vein quality and procedural success may sometimes justify joint placement, particularly for short‐term PIVCs. However, patients should be informed that avoiding joint sites may increase the likelihood of multiple insertion attempts, allowing them to make an informed decision.

Differentiating between *procedural and therapy PIVCs* clarifies how long the catheter will remain, balancing comfort and success.

Engaging patients in discussion about previous experiences and preferences regarding insertion site supports shared decision‐making and aligns with evidence‐based, patient‐centred care.

For *therapy PIVCs*, selecting a comfortable and stable site, such as the forearm, can enhance patient comfort and reduce complications (Drugeon et al. 2024; Buetti et al. 2022).

*In cases of uncertainty*, patients should be informed that the initial site may only meet immediate needs and that another catheter could be required if the treatment plan evolves. This anticipatory communication helps prevent misunderstandings and promotes patient‐centred care.

### 4.4 ACCS 3: Ensure Competency

Competency frameworks for nurses should emphasise first‐attempt success, assessment of DIVA, and the application of aseptic technique. Ongoing education and reflective practice are essential to sustain safe and effective PIVC management. Evidence indicates that PIVCs are best inserted and maintained by trained clinicians adhering to current guidelines (Australian Commission Safety Quality Health Care 2021). The growing range of vascular devices, advancing technology, and high workloads highlight the need to define each clinician's role for safe and efficient vascular access. Differentiating between *procedural and therapy PIVCs* is crucial due to their distinct risks. *Procedural PIVCs* have a low infection risk, and the focus is therefore on insertion success at the first attempt. *Therapy PIVCs* carry higher risks of infections, phlebitis, infiltration and occlusion. Training should focus on the prevention of complications specific to each of the two strategies.

For *procedural PIVCs*, competency frameworks should emphasise first‐attempt success, identification of difficult venous access, and appropriate use of vascular‐access technologies. Adherence to aseptic technique and infection‐prevention standards remains a cornerstone of evidence‐based vascular access care. This focused training minimises immediate complications and ensures that catheters are used safely and efficiently for short‐term needs.

For *therapy PIVCs*, structured training has been shown to be essential, particularly regarding additional securement techniques and the use of standardised equipment such as needleless connectors, extension sets, integrated catheters and flushing techniques, that can prolong functional catheter dwell time and increase therapy completion rates. This approach reduces long‐term complications, such as infections and occlusions, while lowering healthcare costs (Steere et al. 2019). Multimodal training programmes for healthcare providers have demonstrated a significant reduction in catheter‐related bloodstream infections (Bhatt et al. 2021; Saliba et al. 2018; Garcia‐Gasalla et al. 2019). In complex cases, including DIVA patients, those with chronic conditions, or those requiring prolonged therapy, specialised vascular access teams are crucial. These professionals bring expertise in managing challenging insertions, minimising complications (Bell and Spencer 2021; Savage et al. 2019; Whalen et al. 2017), and selecting optimal devices, such as midlines. Integrating such teams into clinical practice has been associated with improved patient outcomes and more efficient vascular access management.

### 4.5 ACCS 4: Choose the Right Insertion Site and PIVC Size

Selecting the right insertion site and catheter size is critical to optimise outcomes and minimise complications, particularly when addressing the distinct needs of *procedural* and *therapy PIVCs*. Current recommendations already emphasise (in general terms) that site selection should be based on intended use and duration; however most PIVCs are inserted in the cubital fossae in EDs and on the hands or cubital fossae in medical wards (Alexandrou et al. 2018; Xu et al. 2023). Data on the effect of site as a risk factor for PIVC complications primarily come from secondary analyses of epidemiological studies or randomised controlled trials (RCTs) with different objectives. Although the results of these studies are consistent, their conclusions require cautious interpretation, and future research is needed to validate these recommendations and refine site‐selection strategies tailored to specific clinical contexts.

*For procedural PIVCs*, site selection should prioritise accessibility and efficiency while preserving proximal veins for future use. Evidence suggests that distal sites, such as the back of the hand, offer an optimal balance between ease of cannulation and protection of vascular capital (van Loon et al. 2016, 2019). Data from Australian EDs support this approach, showing that patients with DIVA are often cannulated at distal sites such as the hand or wrist due to their accessibility (Sweeny et al. 2022). These choices reflect the short‐term purpose of procedural catheters, where immediate usability is valued over long dwell time, but vein fragility and catheter diameter must still be considered, especially for contrast agents (Nickel et al. 2024).

For *therapy PIVCs*, prevention of delayed complications is more important. Dislodgement, infiltration and occlusion risk is higher when the PIVC is placed over a joint where mobility is higher (i.e., hand, wrist or cubital fossae) (Marsh et al. 2020; Drugeon et al. 2024). Some data suggest that forearm insertion raises the risk of phlebitis (Zingg et al. 2023) and interestingly, hands offer protection against CRBSI (Buetti et al. 2022). Overall, to maximise dwell time and minimise complications, the literature supports use of straight, non‐torturous segments of the cephalic, basilic or medial forearm veins, while avoiding flexion areas and, when possible, the dominant side (Nickel et al. 2024).

The choice of the catheter size depends on the infusion rate required and the diameter of the cannulated vein so that the catheter to vein ratio is no more than 30%. It is not based on the expected dwell time. Smaller catheters (< 20 gauge) increase occlusion risk (Zingg et al. 2023) and accidental removal (Wallis et al. 2014).

### 4.6 ACCS 5: Maximise First Insertion Success

Building on the site‐selection principles outlined in ACCS 4, ACCS 5 focuses on the strategies and competencies that enable successful cannulation at the first attempt. Focusing on first‐attempt success through targeted assessments, advanced technologies, and expertise improves outcomes, reduces complications, and builds patient confidence in *procedural and therapy PIVCs*. Success minimises patient anxiety, pain and caregiver workload, as failure rates can reach 40%, leading to complications like multiple puncture attempts, arterial puncture, hematoma and nerve damage (Schults et al. 2022; Jacobson and Winslow 2005; Taşdelen et al. 2024; van Loon, Buise, et al. 2018). Inserter experience is key, guided by predictive criteria (e.g., obesity, vascular history, prior difficult access) using tools like the A‐DIVA scale (Witting 2012; Nickel et al. 2024; Australian Commission Safety Quality Health Care 2021; van Loon et al. 2019; Hallam et al. 2016). For DIVA patients, ultrasound has been shown to significantly improve success in adults, while compact infrared devices demonstrate promise in the paediatric population, although further validation is required (Annalyn et al. 2024; Ray‐Barruel et al. 2024).

For *procedural PIVCs*, where rapid access is required, literature indicates that prioritising first‐attempt success is particularly important. Studies suggest that the back of the hand or wrist may provide easier access and higher success rates, especially in DIVA patients, while avoiding flexion areas is associated with fewer complications (Pittiruti et al. 2023). These sites may be more suitable for novice inserters in patients without DIVA. However, in certain clinical settings such as radiology, EDs and anaesthesia, cubital fossae cannulation remains common and will continue to be widely used due to the need for reliable, high‐flow access for imaging procedures, resuscitation or perioperative care. While these placements carry a higher risk of post‐insertion complications, they are often the most practical option in these contexts (Sweeny et al. 2022; Guenezan et al. 2021).

For *therapy PIVCs*, appropriate and likely more skilled inserters are needed for many patients so the forearm veins can be used with first‐time insertion success. Device choice must always consider the patient's preferences and the balance between benefits and risks. For therapies exceeding 7 days in DIVA patients, midline catheters may represent an appropriate option when compatible with peripheral administration (Timsit et al. 2022). Long peripheral catheters inserted under ultrasound guidance may also be appropriate, especially when targeting deeper peripheral veins. Current evidence indicates that these procedures are most effective when performed by experienced clinicians or vascular access teams with ultrasound guidance (Pittiruti et al. 2023; Timsit et al. 2022).

### 4.7 ACCS 6: Insert and Secure

#### 4.7.1 Skin Antisepsis

Skin disinfection is key to preventing catheter‐related infections by reducing bacterial load at the insertion site. Guidelines recommend the use of 2% Chlorhexidine Gluconate (CHG)‐alcohol thanks to the immediate and potent bactericidal effect of alcohol, and the sustained antimicrobial effect of CHG (Nickel et al. 2024; Australian Commission Safety Quality Health Care 2021; Pittiruti et al. 2023; Bodenham Chair et al. 2016; Capdevila et al. 2016; Baradelle et al. 2016; Queensland Government D of H 2019). Povidone‐iodine (PVI)‐alcohol may be used as an alternative in patients with CHG contraindications or in low‐resource settings, as PVI is less expensive than CHG (World Health Organization 2024). Proper application techniques and drying times further minimise infection risk (Mimoz et al. 2016).

Infection risk varies between *procedural and therapy PIVCs* due to different dwell times. *Procedural PIVCs*, used briefly, require less prolonged antiseptic activity, while *therapy PIVCs*, remaining several days, face higher infection risks, particularly after 48 h. Tailored disinfection strategies optimise protection without overtreatment, reducing unnecessary antiseptic exposure, which may lead to allergies, bacterial resistance or antibiotic cross‐resistance, though these risks are debated (Drugeon, Rickard, Buetti, et al. 2025; Drugeon, Rickard, Schults, et al. 2025; Kampf 2016).

For *procedural PIVCs*, which are intended for short‐term use, thorough skin disinfection at insertion is essential; extended antiseptic action is less critical. Some studies indicate that alcohol or sodium hypochlorite alone may provide adequate rapid bactericidal action and can be more cost‐effective than alcohol‐based formulations of CHG or PVI (Drugeon et al. 2022; Maiwald and Chan 2012). Sodium hypochlorite could be an alternative for patients intolerant to alcohol, though its efficacy remains under‐studied (Drugeon et al. 2022; Forni et al. 2015).

For *therapy PIVCs*, used for prolonged therapies, maintaining antiseptic activity at the insertion site with > 0.5% CHG in alcohol (unless contraindicated) has been associated with lower infection rates and is widely recommended in guidelines (Mermel 2017).

#### 4.7.2 Equipment

Advances in vascular access equipment, such as integrated catheters, impregnated caps and needleless valves, aim to reduce complications but increase costs and do not all have strong evidence. The choice of equipment must be adapted to the patient's clinical needs and intended duration of catheter use to balance safety and cost‐effectiveness.

For *procedural PIVCs*, simplicity and cost‐efficiency are generally prioritised due to the lower risk of delayed complications (CRBSI, local infection, phlebitis). However, in response to certain environmental constraints, particularly in prehospital care, integrated catheters offer significant advantages when the patient needs vascular access without a line. These all‐in‐one devices combine inbuilt extension tubing, needleless connectors, and a stabilising base into a single, closed system. This preassembled design is immediately ready for use in emergencies and reduces handling compared to the traditional setup of a separate catheter, extension tubing and valve. By minimising assembly steps, integrated catheters lower the risk of soiling and, unlike standard equipment connected to an infusion line, are less prone to accidental dislodgement during patient transfers, particularly during stretcher movements. In prehospital settings where rapid interventions and repeated medication administration, such as analgesia or sedation, are required, these devices can provide practical benefits by ensuring stable vascular access throughout transport. However, their routine use should be carefully weighed against cost and necessity, as their advantages may be context‐dependent.

For *therapy PIVCs*, devices should support prolonged catheter dwell time while minimising delayed complications. Integrated catheters are likely cost‐effective in this scenario due to their secure design, contributing to longer dwell times and reduced phlebitis and occlusion (González López et al. 2014; Rickard et al. 2023). Additionally, bundled approaches combining integrated catheters with needleless valves, impregnated caps, and pre‐filled syringes further reduce mechanical complications and costs (Guenezan et al. 2021). The cost savings from these strategies can be reinvested in staff training, prevention programmes and innovative technologies (Gidaro et al. 2024; Ray‐Barruel et al. 2019; Mimoz et al. 2024). Further research is needed to standardise bundle components and validate their clinical and economic benefits.

#### 4.7.3 Dressing

Inadequate PIVC fixation can cause displacement, premature removal, treatment interruption and promote infections. Semi‐permeable polyurethane dressings, simple or bordered, and integrated securement devices with transparent windows and fabric are recommended for stability and insertion site visibility (Nickel et al. 2024; Australian Commission Safety Quality Health Care 2021; Rickard et al. 2018; Pittiruti et al. 2023; Chaize and Lepelletier 2019; Marsh et al. 2017). Secondary supports like sutureless systems, tissue adhesive and strips enhance fixation. While sterile gauze has limited evidence, RCTs suggest fewer complications, possibly from reduced visibility issues (Corley et al. 2019). CHG‐impregnated dressings lower infection risks in high‐risk patients requiring central and arterial catheters, but their impact with PIVCs is underexplored (Ullman et al. 2015; Timsit et al. 2012; Biehl et al. 2016). Further research is needed to determine whether CHG‐impregnated dressings provide similar benefits for PIVCs, particularly in patients at increased risk of infection.

For *procedural PIVCs*, available evidence suggests that inexpensive transparent polyurethane dressings are generally adequate, given the short duration of use and lower risks of displacement or infection (Rickard et al. 2018). These dressings provide adequate site visibility and basic stabilisation for short‐term use. Many patients with *procedural PIVCs* are sedated or bed bound for short procedures or assessments, reducing the risks of dislodgement. More advanced fixation systems are generally unnecessary, though in certain environments, such as prehospital care or emergency procedures, slightly reinforced dressings may be considered to secure the catheter during patient transport.

For *therapy PIVCs*, longer catheter dwell times justify the use of more secure fixation methods. Integrated securement devices or tissue adhesives can help reduce the risk of catheter displacement, particularly in patients with DIVA, frequent hospital admissions or complex chronic conditions. However, evidence comparing advanced fixation systems with simple dressings remains inconsistent, with no clear advantage demonstrated in preventing complications in adults (Rickard et al. 2018). One study has shown the superiority of a central venous catheter (CVC) anchoring device in children; however, to our knowledge, no similar study has been conducted with PIVCs in adults (Kleidon et al. 2024). Consequently, simple polyurethane dressings remain a cost‐effective and appropriate choice for most patients. Further research is required to determine the optimal dressing strategies for prolonged PIVC use.

### 4.8 ACCS 7: Document Decisions and Care

Nurses are responsible for ongoing assessment, documentation and timely removal of PIVCs, making nursing vigilance a cornerstone of device safety and infection prevention. Accurate documentation of PIVC insertion, maintenance, and removal is essential for continuity and quality of care, enhancing patient safety by enabling the tracing of complications and their management (Australian Commission Safety Quality Health Care 2021; Bodenham Chair et al. 2016; Capdevila et al. 2016). Digital tools or barcode systems simplify this process, facilitate audits and reduce errors, improving standardisation. However, documentation is incomplete or absent in 20%–50% of cases (Alexandrou et al. 2018; Aghdassi et al. 2019; Berger et al. 2022; Høvik et al. 2020; Ray‐Barruel et al. 2023), compromising care quality and the ability to track complications. Strengthening adherence to documentation is thus critical.

For *procedural PIVCs*, which are generally used for short periods and involve fewer caregivers, the literature suggests that simplified approaches such as checklists focusing on insertion details, use, adverse events, and planned removal may be sufficient (Capdevila et al. 2016).

For *therapy PIVCs*, where use is prolonged and multiple caregivers are involved, more comprehensive documentation is typically required. This may include details on insertion (date, time, location, inserter), maintenance (flushing, dressing changes), and removal (date, reason) as well as ongoing assessment of complications. Daily documentation of the PIVC need or removal plan is important. A standardised digital form ensures systematic capture of this information. Tailoring documentation practices to *procedural and therapy PIVCs* ensures practicality without sacrificing safety or traceability (Capdevila et al. 2016).

### 4.9 ACCS 8: Routine Use: Inspect, Access and Flush

It is advisable to monitor the puncture site at regular intervals for infection, phlebitis, leakage, and to check the integrity of the dressing and function of the catheter. Injection sites, such as taps and valves, should be handled with standard hygiene precautions, including disinfection of the ports with alcohol with adequate dry time. Additionally, saline flushes should be performed at regular intervals, although the frequency is not defined, and after each drug administration, to reduce the risk of occlusion and avoid mixing incompatible drugs (Nickel et al. 2024; Australian Commission Safety Quality Health Care 2021; Pittiruti et al. 2023). Differentiating between *procedural and therapy PIVCs* ensures practices are aligned with the specific risks and duration of catheter use.

For *procedural PIVCs*, the need for routine surveillance is minimal. However, monitoring should prioritise detecting insertion failures or immediate complications, such as dislodgement or infiltration, as these are the most relevant risks for short‐term use.

For *therapy PIVCs*, more frequent and thorough monitoring is required, necessitating greater staff involvement over time. Particular attention has been recommended for early detection of infection, as timely intervention is critical in preventing progression to severe complications.

### 4.10 ACCS 9 and 10: Review Ongoing Need, Remove Safely and Replace if Needed

PIVCs must be reassessed daily to ensure their continued necessity or to prompt removal. Regular review and documentation minimise the risk of unnecessary catheter retention, reduce complications and optimise patient care (Australian Commission Safety Quality Health Care 2021; Mimoz et al. 2024).

For *procedural PIVCs*, reassessment is straightforward. Once their dedicated purpose is completed, the catheter should be promptly removed. Clear communication with the patient, the clinical team and documentation of this change are essential to ensure appropriate ongoing care if the treatment plan evolves and further vascular access is required (Australian Commission Safety Quality Health Care 2021).

For *therapy PIVCs*, reassessment is more complex due to their longer duration of use. The I‐DECIDED decision framework developed from evidence‐based recommendations, has been proposed as a structured approach to guide this process. This mnemonic‐based tool facilitates reassessment, reduces unnecessary catheter use, and supports improved documentation (Ray‐Barruel et al. 2023). Additionally, PIVCs inserted during night shifts or in uncontrolled environments, such as prehospital care, require systematic review by the day team. This should assess whether asepsis was maintained during insertion, consider their continued necessity, and prompt appropriate removal and replacement. Such reviews are critical to maintaining catheter safety and optimising patient care.

## 5 Discussion

### 5.1 Expected Benefits and Clinical Impact

In recent years, the understanding of the multiple and complex factors involved in PIVC complications has developed, but to date guidelines and policies treat all PIVCs equally. The distinction between *procedural and therapy PIVCs* introduces a practical and adaptable framework that is easily applied at the bedside and seamlessly integrated into daily clinical practice. This framework reinforces nurses' pivotal role in vascular access safety and highlights the need for nursing leadership in implementing evidence‐based PIVC practices. Its simplicity and repeatability make it an effective tool for improving vascular access management across diverse settings. Importantly, this model respects and supports clinicians' autonomy by enabling personalised, patient‐centred decision‐making. Patients value care that is individualised rather than rigidly tied to protocols, and this framework empowers healthcare professionals to adapt their choices to specific clinical needs while maintaining the highest standards of safety and effectiveness.

By adapting policies to real‐world clinical environments, the *procedural and therapy PIVC* framework overcomes the rigidity of one‐size‐fits‐all approaches. Strict adherence to rigid protocols can cause delays, failed attempts and dissatisfaction (Xu et al. 2023). This model promotes flexibility, enabling care tailored to the patient's condition and the catheter's intended duration.

Cost‐effectiveness is another key advantage. For *procedural PIVCs*, simple, affordable materials reduce waste, freeing resources for advanced technologies like integrated catheters or even antimicrobial dressings for therapy PIVCs, where stability and reduced complications are essential. This strategic allocation improves outcomes, prolongs catheter dwell times, and enhances patient satisfaction. All these elements are summarised in Table 2.

**TABLE 2:** Main recommendations for procedural and therapy PIVCs.

	Procedural PIVCs	Tehrapy PIVCs
Expected dwell time	< 24 h	≥ 24 h and < 7 days
Need	Avoid « just in case »	Consider midline if need > 7 days and/or DIVA criteria
Inform and partner with patient	Fully informed	
Competency	Insertion training	Guidelines and multimodal interventions for both insertion and post‐insertion management
Insertion site	Hand (prefer)/wrist/ACF (last preference)	Forearm (prefer)/upper arm
Success at the first attempt	Assessment for DIVA status Consider inserter expertise and confidence Ultrasound use for DIVA preferred Vascular Access Advanced Inserter for DIVA preferred	
Skin disinfection	Alcohol alone/Aqueous sodium hypochlorite	2% chlorhexidine‐alcohol
Bundle	Easy to use and adapted to the indication	Bundle of high‐performance devices and procedures
Dressing	Simple polyurethane dressing	
		Consider additional securement (gum mastic, tissue adhesive, antimicrobial dressing if immunosuppressed)
Documentation	Checklist of insertion detail and use including order to remove < 24 h	Exhaustive and standardised data set including daily review of need/plan for removal
Routine use	Monitor insertion site/flush	
Reassessment of need	Consider another PIVC if need changes to > 24 h	Use the I‐DECIDED rule

*Note:* See van Loon et al. (2019) and Paterson et al. (2022).

Abbreviations: DIVA, difficult intravenous access; PIVC, peripheral intravenous catheter.

At the same time, this review also highlighted important evidence gaps, particularly regarding *procedural PIVCs*, simplified fixation methods, and patient‐reported outcomes such as comfort and sleep disturbance. These gaps underline the need for further empirical research to strengthen the evidence base supporting this framework.

By balancing simplicity for short‐term use with innovation for prolonged therapies, this model optimises resources, improves safety, and fosters a more sustainable healthcare system, delivering the adaptability and quality modern patients and clinicians expect.

### 5.2 Strengths and Limitations

The main strength of this work lies in its novel attempt to differentiate between *procedural and therapy PIVCs* through a structured concept analysis supported by a narrative review. By integrating diverse sources of evidence, including clinical studies, international guidelines, consensus statements and professional society position papers, and by structuring the synthesis according to the ACCS, this review provides a pragmatic and patient‐centred framework that addresses an important gap in current practice. Furthermore, we followed the SANRA quality criteria, applied a predefined search strategy in PubMed, and complemented it with snowballing to minimise the risk of missing relevant literature.

However, we acknowledge several limitations of this methodological approach. As a narrative review, the process does not guarantee the same level of comprehensiveness or critical appraisal as a systematic review. Despite using predefined search terms, snowballing from guideline references, and consulting the authors' reference collections, the possibility of missing relevant publications cannot be excluded. In addition, no formal quality appraisal of included studies was undertaken, consistent with the aims of a narrative review but limiting the strengths of the evidence base. Finally, the synthesis was interpretative rather than quantitative, reflecting the exploratory nature of this work. Some statements within the framework are not directly supported by studies; these reflect reasoned reflections of the authors, derived from the synthesis of all available evidence and the conceptual distinction between *procedural and therapy PIVCs*. They should therefore be interpreted as hypothesis‐generating rather than prescriptive recommendations. These limitations are inherent to narrative reviews and reinforce that our proposed taxonomy should be considered an early conceptual framework requiring further validation.

## 6 Conclusion

Adopting a *procedural and therapy PIVC* model optimises implementation, benefiting patients, staff and healthcare facilities. Institutions and clinicians must embrace this framework, adapt policies, and invest in expert staff. Further research on short‐term complications and evidence‐based tools for DIVA patients is essential to refine practices and improve outcomes globally. Importantly, this work should be understood as exploratory conceptual proposals rather than prescriptive guidelines, and the framework requires empirical testing and validation before being considered for formal implementation.

## Author Contributions

B.D., C.M.R., O.M. made substantial contributions to conception and design, or acquisition of data or analysis and interpretation of data; B.D., C.M.R., O.M., V.C., N.P. involved in drafting the manuscript or revising it critically for important intellectual content and given final approval of the version to be published. Each author should have participated sufficiently in the work to take public responsibility for appropriate portions of the content; B.D. agreed to be accountable for all aspects of the work in ensuring that questions related to the accuracy or integrity of any part of the work are appropriately investigated and resolved.

## Ethics Statement

The authors have nothing to report.

## Conflicts of Interest

O.M., B.D. received funding for congress attendance, and research funding from Becton Dickinson and 3 M/Solventum. C.M.R.'s employer (University of Queensland or Griffith University) received unrestricted investigator‐initiated research on her behalf from B.D., 3M/Solventum and Cardinal Health and consultancy payments for lectures or expert opinion from 3M, BBraun and ITL Biomedical. C.M.R. is co‐director of AVATAR (Griffith University) which received educational grants from 3M/Solventum, Angiodynamics, Spectrum Vascular and ICU Medical, V.C. and N.P. have no conflicts of interest.

## Supporting information


**Data S1:** Research strategy.

## Data Availability

Data sharing not applicable to this article as no datasets were generated or analyzed during the current study.

## References

[jan70347-bib-0001] Aghdassi, S. J. S. , C. Schröder , D. Gruhl , P. Gastmeier , and F. Salm . 2019. “Point Prevalence Survey of Peripheral Venous Catheter Usage in a Large Tertiary Care University Hospital in Germany.” Antimicrobial Resistance and Infection Control 8, no. 1: 15. 10.1186/s13756-019-0468-8. 30675342 PMC6335674

[jan70347-bib-0002] Alexandrou, E. , G. Ray‐Barruel , P. J. Carr , et al. 2015. “International Prevalence of the Use of Peripheral Intravenous Catheters: Prevalence of the Use of PIVCs.” Journal of Hospital Medicine 10, no. 8: 530–533. 10.1002/jhm.2389. 26041384

[jan70347-bib-0003] Alexandrou, E. , G. Ray‐Barruel , P. J. Carr , et al. 2018. “Use of Short Peripheral Intravenous Catheters: Characteristics, Management, and Outcomes Worldwide.” Journal of Hospital Medicine 13, no. 5: 3039. 10.12788/jhm.3039. 29813140

[jan70347-bib-0004] Annalyn, N. S. L. , X. R. G. Leow , W. W. Ang , and Y. Lau . 2024. “Effectiveness of Near‐Infrared Light Devices for Peripheral Intravenous Cannulation in Children and Adolescents: A Meta‐Analysis of Randomized Controlled Trials.” Journal of Pediatric Nursing 75: e81–e92. 10.1016/j.pedn.2023.12.034. 38195374

[jan70347-bib-0005] Australian Commission Safety Quality Health Care . 2021. Management of Peripheral Intravenous Catheters Clinical Care Standard. Australian Commission on Safety & Quality in Health Care.

[jan70347-bib-0006] Baethge, C. , S. Goldbeck‐Wood , and S. Mertens . 2019. “SANRA—A Scale for the Quality Assessment of Narrative Review Articles.” Research Integrity and Peer Review 4, no. 1: 5. 10.1186/s41073-019-0064-8. 30962953 PMC6434870

[jan70347-bib-0007] Baradelle, O. , J. Fabry , V. Surville , H. Boulestreau , and N. Sanlaville . 2016. “Antisepsie de la Peau Saine Avant Un Geste Invasif Chez L'adulte: Recommandations Pour la Pratique Clinique.” Société Française d'Hygiène Hospitalière 24, no. 2: 192.

[jan70347-bib-0008] Bell, J. A. , and T. R. Spencer . 2021. “Implementing an Emergency Department Vascular Access Team: A Quality Review of Training, Competency, and Outcomes.” Journal of Vascular Access 22, no. 1: 81–89. 10.1177/1129729820924554. 32484002

[jan70347-bib-0009] Berger, S. , K. Winchester , R. B. Principe , and E. Culverwell . 2022. “Prevalence of Peripheral Intravenous Catheters and Policy Adherence: A Point Prevalence in a Tertiary Care University Hospital.” Journal of Clinical Nursing 31, no. 15–16: 2324–2330. 10.1111/jocn.16051. 34535927

[jan70347-bib-0010] Bhatt, C. R. , R. Meek , C. Martin , et al. 2021. “Effect of Multimodal Interventions on Peripheral Intravenous Catheter–Associated *Staphylococcus aureus* Bacteremia and Insertion Rates: An Interrupted Time‐Series Analysis.” Academic Emergency Medicine 28, no. 8: 909–912. 10.1111/acem.14225. 33529465

[jan70347-bib-0011] Biehl, L. M. , A. Huth , J. Panse , et al. 2016. “A Randomized Trial on Chlorhexidine Dressings for the Prevention of Catheter‐Related Bloodstream Infections in Neutropenic Patients.” Annals of Oncology 27, no. 10: 1916–1922. 10.1093/annonc/mdw275. 27456299

[jan70347-bib-0012] Bodenham Chair, A. , S. Babu , J. Bennett , et al. 2016. “Association of Anaesthetists of Great Britain and Ireland: Safe Vascular Access 2016.” Anaesthesia 71, no. 5: 573–585. 10.1111/anae.13360. 26888253 PMC5067617

[jan70347-bib-0013] Buetti, N. , M. Abbas , D. Pittet , et al. 2022. “Lower Risk of Peripheral Venous Catheter‐Related Bloodstream Infection by Hand Insertion.” Antimicrobial Resistance and Infection Control 11, no. 1: 80. 10.1186/s13756-022-01117-8. 35659775 PMC9164319

[jan70347-bib-0014] Capdevila, J. A. , M. Guembe , J. Barberán , et al. 2016. “2016 Expert Consensus Document on Prevention, Diagnosis and Treatment of Short‐Term Peripheral Venous Catheter‐Related Infections in Adults.” Cirugía Cardiovascular 23, no. 4: 192–198. 10.1016/j.circv.2016.06.001. 27580009

[jan70347-bib-0015] Carr, P. J. , J. C. R. Rippey , C. A. Budgeon , M. L. Cooke , N. Higgins , and C. M. Rickard . 2016. “Insertion of Peripheral Intravenous Cannulae in the Emergency Department: Factors Associated With First‐Time Insertion Success.” Journal of Vascular Access 17, no. 2: 182–190. 10.5301/jva.5000487. 26660037

[jan70347-bib-0016] Chaize, P. , and D. Lepelletier . 2019. “Prévention des Infections Liées aux Cathéters Périphériques Vasculaires et Sous‐Cutanés.” Société Française d'Hygiène Hospitalière 27, no. 2: 23.

[jan70347-bib-0017] Corley, A. , A. J. Ullman , G. Mihala , G. Ray‐Barruel , E. Alexandrou , and C. M. Rickard . 2019. “Peripheral Intravenous Catheter Dressing and Securement Practice Is Associated With Site Complications and Suboptimal Dressing Integrity: A Secondary Analysis of 40,637 Catheters.” International Journal of Nursing Studies 100: 103409. 10.1016/j.ijnurstu.2019.103409. 31629208

[jan70347-bib-0018] Drugeon, B. , J. Guenezan , M. Pichon , et al. 2023. “Incidence, Complications, and Costs of Peripheral Venous Catheter‐Related Bacteraemia: A Retrospective, Single‐Centre Study.” Journal of Hospital Infection 135: 67–73. 10.1016/j.jhin.2023.02.012. 36918069

[jan70347-bib-0019] Drugeon, B. , N. Marjanovic , M. Boisson , N. Buetti , O. Mimoz , and J. Guenezan . 2024. “Insertion Site and Risk of Peripheral Intravenous Catheter Colonization and/or Local Infection: A Post Hoc Analysis of the CLEAN 3 Study Including More Than 800 Catheters.” Antimicrobial Resistance and Infection Control 13, no. 1: 57. 10.1186/s13756-024-01414-4. 38840171 PMC11151591

[jan70347-bib-0020] Drugeon, B. , M. Pichon , N. Marjanovic , et al. 2022. “Peripheral Venous Catheter Colonization After Skin Disinfection With 0.5% Aqueous Sodium Hypochlorite, Preceded or Not by One Application of 70% Ethanol (DACLEAN): A Single‐Centre, Randomized, Open‐Label, Pilot Study.” Journal of Hospital Infection 120: 123–126. 10.1016/j.jhin.2021.11.012. 34822950

[jan70347-bib-0021] Drugeon, B. , C. M. Rickard , N. Buetti , J. Guenezan , and O. Mimoz . 2025. “Myths Glorify What Reality Neglects: Efficacy and Safety of Chlorhexidine Gluconate.” Current Infectious Disease Reports 27, no. 1: 14. 10.1007/s11908-025-00865-z.

[jan70347-bib-0022] Drugeon, B. , C. M. Rickard , J. A. Schults , J. Guenezan , and O. Mimoz . 2025. “Chlorhexidine and Povidone‐Iodine: Unmasking the Unknown in Catheter‐Related Infection Prevention, a Narrative Review.” Journal of Infection and Public Health 18, no. 9: 102851. 10.1016/j.jiph.2025.102851. 40479965

[jan70347-bib-0023] Egerton‐Warburton, D. , F. McAllan , R. Ramanan , et al. 2019. “Human Factor‐Designed Multimodal Intervention Reduces the Rate of Unused Peripheral Intravenous Cannula Insertion.” Emergency Medicine Australasia 31, no. 3: 372–377. 10.1111/1742-6723.13165. 30208510

[jan70347-bib-0024] Evison, H. , A. Sweeny , J. Ranse , et al. 2021. “Idle Peripheral Intravenous Cannulation: An Observational Cohort Study of Pre‐Hospital and Emergency Department Practices.” Scandinavian Journal of Trauma, Resuscitation and Emergency Medicine 29, no. 1: 126. 10.1186/s13049-021-00941-y. 34454555 PMC8403444

[jan70347-bib-0025] Forni, C. , T. Sabattini , F. D'Alessandro , et al. 2015. “Use of Sodium Hypochlorite for Skin Antisepsis Before Inserting a Peripheral Venous Catheter: A Pilot Study.” Biological Research for Nursing 17, no. 3: 330. 10.1177/1099800414545509. 25230748

[jan70347-bib-0026] Garcia‐Gasalla, M. , M. Arrizabalaga‐Asenjo , C. Collado‐Giner , et al. 2019. “Results of a Multi‐Faceted Educational Intervention to Prevent Peripheral Venous Catheter‐Associated Bloodstream Infections.” Journal of Hospital Infection 102, no. 4: 449–453. 10.1016/j.jhin.2019.02.004. 30771370

[jan70347-bib-0027] Gidaro, A. , M. Quici , D. Giustivi , et al. 2024. “Integrated Short Peripheral Intravenous Cannulas and Risk of Catheter Failure: A Systematic Review and Meta‐Analysis.” Journal of Vascular Access 26, no. 2: 372–380. 10.1177/11297298231218468. 38166435

[jan70347-bib-0028] González López, J. L. , A. Arribi Vilela , E. Fernández Del Palacio , J. Olivares Corral , C. Benedicto Martí , and P. Herrera Portal . 2014. “Indwell Times, Complications and Costs of Open vs. Closed Safety Peripheral Intravenous Catheters: A Randomized Study.” Journal of Hospital Infection 86, no. 2: 117–126. 10.1016/j.jhin.2013.10.008. 24373830

[jan70347-bib-0029] Guenezan, J. , N. Marjanovic , B. Drugeon , et al. 2021. “Chlorhexidine Plus Alcohol Versus Povidone Iodine Plus Alcohol, Combined or Not With Innovative Devices, for Prevention of Short‐Term Peripheral Venous Catheter Infection and Failure (CLEAN 3 Study): An Investigator‐Initiated, Open‐Label, Single Centre, Randomised‐Controlled, Two‐By‐Two Factorial Trial.” Lancet Infectious Diseases 21, no. 7: 1038–1048. 10.1016/S1473-3099(20)30738-6. 33539734

[jan70347-bib-0030] Hadaway, L. 2012. “Short Peripheral Intravenous Catheters and Infections: Journal of Infusion Nursing.” Journal of Infusion Nursing 35, no. 4: 230–240. 10.1097/NAN.0b013e31825af099. 22759827

[jan70347-bib-0031] Hallam, C. , V. Weston , A. Denton , et al. 2016. “Development of the UK Vessel Health and Preservation (VHP) Framework: A Multi‐Organisational Collaborative.” Journal of Infection Prevention 17, no. 2: 65–72. 10.1177/1757177415624752. 28989456 PMC5074217

[jan70347-bib-0032] Hawkins, T. , J. H. Greenslade , J. Suna , et al. 2018. “Peripheral Intravenous Cannula Insertion and Use in the Emergency Department: An Intervention Study.” Academic Emergency Medicine 25, no. 1: 26–32. 10.1111/acem.13335. 29044739

[jan70347-bib-0033] Helm, R. E. , J. D. Klausner , J. D. Klemperer , L. M. Flint , and E. Huang . 2015. “Accepted but Unacceptable: Peripheral IV Catheter Failure.” Journal of Infusion Nursing 38, no. 3: 189–203. 10.1097/NAN.0000000000000100. 25871866

[jan70347-bib-0034] Høvik, L. H. , K. H. Gjeilo , and S. Lydersen . 2020. “Use of Peripheral Venous Catheters in Two Norwegian Hospitals.” Tidsskrift for Den Norske Legeforening 140: 2324. 10.4045/tidsskr.19.0653 32463202

[jan70347-bib-0035] Jacobson, A. F. , and E. H. Winslow . 2005. “Variables Influencing Intravenous Catheter Insertion Difficulty and Failure: An Analysis of 339 Intravenous Catheter Insertions.” Heart & Lung 34, no. 5: 345–359. 10.1016/j.hrtlng.2005.04.002. 16157191

[jan70347-bib-0036] Kampf, G. 2016. “Acquired Resistance to Chlorhexidine—Is It Time to Establish an ‘Antiseptic Stewardship’ Initiative?” Journal of Hospital Infection 94, no. 3: 213–227. 10.1016/j.jhin.2016.08.018. 27671220

[jan70347-bib-0037] Kleidon, T. M. , J. Schults , V. Gibson , et al. 2024. “Securement to Prevent Noncuffed Central Venous Catheter Dislodgement in Pediatrics: The SECURED Superiority Randomized Clinical Trial.” JAMA Pediatrics 178, no. 9: 861–869. 10.1001/jamapediatrics.2024.2202. 39008311 PMC11250368

[jan70347-bib-0038] Lapostolle, F. , J. Catineau , B. Garrigue , et al. 2007. “Prospective Evaluation of Peripheral Venous Access Difficulty in Emergency Care.” Intensive Care Medicine 33, no. 8: 1452–1457. 10.1007/s00134-007-0634-y. 17554524

[jan70347-bib-0039] Larsen, E. , S. Keogh , N. Marsh , and C. Rickard . 2017. “Experiences of Peripheral IV Insertion in Hospital: A Qualitative Study.” British Journal of Nursing 26, no. 19: S18–S25. 10.12968/bjon.2017.26.19.S18. 29068742

[jan70347-bib-0040] Lim, S. , G. Gangoli , E. Adams , et al. 2019. “Increased Clinical and Economic Burden Associated With Peripheral Intravenous Catheter–Related Complications: Analysis of a US Hospital Discharge Database.” Inquiry 56: 1–14. 10.1177/0046958019875562. PMC6747868 31524024

[jan70347-bib-0041] Lim, Z. J. , D. Nagle , F. McAllan , et al. 2020. “Evaluating the Sustained Effectiveness of a Multimodal Intervention Aimed at Influencing PIVC Insertion Practices in the Emergency Department.” Emergency Medicine Journal 37, no. 7: 444–449. 10.1136/emermed-2019-208852. 32414709

[jan70347-bib-0042] Mailhe, M. , C. Aubry , P. Brouqui , et al. 2020. “Complications of Peripheral Venous Catheters: The Need to Propose an Alternative Route of Administration.” International Journal of Antimicrobial Agents 55, no. 3: 105875. 10.1016/j.ijantimicag.2020.105875. 31926285

[jan70347-bib-0043] Maiwald, M. , and E. S. Y. Chan . 2012. “The Forgotten Role of Alcohol: A Systematic Review and Meta‐Analysis of the Clinical Efficacy and Perceived Role of Chlorhexidine in Skin Antisepsis.” PLoS One 7, no. 9: e44277. 10.1371/journal.pone.0044277. 22984485 PMC3434203

[jan70347-bib-0044] Marsh, N. , E. N. Larsen , A. J. Ullman , et al. 2024. “Peripheral Intravenous Catheter Infection and Failure: A Systematic Review and Meta‐Analysis.” International Journal of Nursing Studies 151: 104673. 10.1016/j.ijnurstu.2023.104673. 38142634

[jan70347-bib-0045] Marsh, N. , J. Webster , G. Mihala , and C. M. Rickard . 2017. “Devices and Dressings to Secure Peripheral Venous Catheters: A Cochrane Systematic Review and Meta‐Analysis.” International Journal of Nursing Studies 67: 12–19. 10.1016/j.ijnurstu.2016.11.007. 27889585

[jan70347-bib-0046] Marsh, N. , J. Webster , A. J. Ullman , et al. 2020. “Peripheral Intravenous Catheter Non‐Infectious Complications in Adults: A Systematic Review and Meta‐Analysis.” Journal of Advanced Nursing 76, no. 12: 3346–3362. 10.1111/jan.14565. 33016412

[jan70347-bib-0047] Maunoury, F. , B. Drugeon , M. Boisson , N. Marjanovic , R. Couvreur , and O. Mimoz . 2022. “Cost‐Effectiveness Analysis of Bundled Innovative Devices Versus Standard Approach in the Prevention of Unscheduled Peripheral Venous Catheters Removal due to Complications in France.” PLoS One 17, no. 6: e0269750. 10.1371/journal.pone.0269750. 35700207 PMC9197036

[jan70347-bib-0048] Mermel, L. A. 2017. “Short‐Term Peripheral Venous Catheter–Related Bloodstream Infections: A Systematic Review.” Clinical Infectious Diseases 65, no. 10: 1757–1762. 10.1093/cid/cix562. 29020252

[jan70347-bib-0049] Mimoz, O. , V. Chopra , and A. Widmer . 2016. “What's New in Skin Antisepsis for Short‐Term Intravascular Catheters: New Data to Address Old Problems?” Intensive Care Medicine 42, no. 12: 2043–2045. 10.1007/s00134-016-4490-5. 27515157

[jan70347-bib-0050] Mimoz, O. , A. Debonne , A. Glanard , O. Keita Perse , and J. C. Lucet . 2024. “Best Practice in the Use of Peripheral Venous Catheters: A Consensus From French Experts.” Infectious Diseases Now 54, no. 5: 104923. 10.1016/j.idnow.2024.104923. 38759732

[jan70347-bib-0051] Morgan, R. , E. Callander , L. Cullen , et al. 2022. “From Little Things, Big Things Grow: An Exploratory Analysis of the National Cost of Peripheral Intravenous Catheter Insertion in Australian Adult Emergency Care.” Emergency Medicine Australasia 34, no. 6: 877–883. 10.1111/1742-6723.14009. 35567373 PMC9790706

[jan70347-bib-0052] Moureau, N. , and V. Chopra . 2016. “Indications for Peripheral, Midline and Central Catheters: Summary of the MAGIC Recommendations.” British Journal of Nursing 25, no. 8: S15–S24. 10.12968/bjon.2016.25.8.S15. 27126759

[jan70347-bib-0053] Nickel, B. , L. Gorski , T. Kleidon , et al. 2024. Infusion Therapy Standards of Practice. Infusion Nurses Society.

[jan70347-bib-0054] Noel, F. , P. Hoang , J. Truchot , A. S. Bard , Y. Yordanov , and P. C. Thiebaud . 2022. “An Educational Intervention to Reduce Unjustified Peripheral Intravenous Infusions in the Emergency Department.” Internal and Emergency Medicine 17, no. 4: 1225–1227. 10.1007/s11739-021-02896-5. 34800237 PMC8605468

[jan70347-bib-0055] Paterson, R. S. , J. A. Schults , E. Slaughter , et al. 2022. “Review Article: Peripheral Intravenous Catheter Insertion in Adult Patients With Difficult Intravenous Access: A Systematic Review of Assessment Instruments, Clinical Practice Guidelines and Escalation Pathways.” Emergency Medicine Australasia 34, no. 6: 862–870. 10.1111/1742-6723.14069. 36038953 PMC9804581

[jan70347-bib-0056] Pittiruti, M. , T. Van Boxtel , G. Scoppettuolo , et al. 2023. “European Recommendations on the Proper Indication and Use of Peripheral Venous Access Devices (The ERPIUP Consensus): A WoCoVA Project.” Journal of Vascular Access 24, no. 1: 165–182. 10.1177/11297298211023274. 34088239

[jan70347-bib-0057] Queensland Government D of H . 2019. “Guideline: Peripheral Intravenous Catheter (PIVC).” https://www.health.qld.gov.au.

[jan70347-bib-0058] Ray‐Barruel, G. 2022. “I‐DECIDED—A Decision Tool for Assessment and Management of Invasive Devices in the Hospital Setting.” British Journal of Nursing 31, no. 8: S37–S43. 35439078 10.12968/bjon.2022.31.8.S37

[jan70347-bib-0059] Ray‐Barruel, G. , V. Chopra , P. Fulbrook , et al. 2023. “The Impact of a Structured Assessment and Decision Tool (I‐DECIDED) on Improving Care of Peripheral Intravenous Catheters: A Multicenter, Interrupted Time‐Series Study.” International Journal of Nursing Studies 148: 104604. 10.1016/j.ijnurstu.2023.104604. 37801935

[jan70347-bib-0060] Ray‐Barruel, G. , M. Cooke , M. Mitchell , V. Chopra , and C. M. Rickard . 2018. “Implementing the I‐DECIDED Clinical Decision‐Making Tool for Peripheral Intravenous Catheter Assessment and Safe Removal: Protocol for an Interrupted Time‐Series Study.” BMJ Open 8, no. 6: e021290. 10.1136/bmjopen-2017-021290. PMC5988165 29866733

[jan70347-bib-0061] Ray‐Barruel, G. , P. Pather , J. A. Schults , and C. M. Rickard . 2024. “Handheld Ultrasound Devices for Peripheral Intravenous Cannulation: A Scoping Review.” Journal of Infusion Nursing 47, no. 2: 75–95. 10.1097/NAN.0000000000000540. 38422403

[jan70347-bib-0062] Ray‐Barruel, G. , H. Xu , N. Marsh , M. Cooke , and C. M. Rickard . 2019. “Effectiveness of Insertion and Maintenance Bundles in Preventing Peripheral Intravenous Catheter‐Related Complications and Bloodstream Infection in Hospital Patients: A Systematic Review.” Infection, Disease & Health 24, no. 3: 152–168. 10.1016/j.idh.2019.03.001. 31005606

[jan70347-bib-0063] Rickard, C. M. , E. Larsen , R. M. Walker , et al. 2023. “Integrated Versus Nonintegrated Peripheral Intravenous Catheter in Hospitalized Adults (OPTIMUM): A Randomized Controlled Trial.” Journal of Hospital Medicine 18, no. 1: 21–32. 10.1002/jhm.12995. 36372995 PMC10099685

[jan70347-bib-0064] Rickard, C. M. , N. Marsh , J. Webster , et al. 2018. “Dressings and Securements for the Prevention of Peripheral Intravenous Catheter Failure in Adults (SAVE): A Pragmatic, Randomised Controlled, Superiority Trial.” Lancet 392, no. 10145: 419–430. 10.1016/S0140-6736(18)31380-1. 30057103

[jan70347-bib-0065] Saliba, P. , A. Hornero , G. Cuervo , et al. 2018. “Interventions to Decrease Short‐Term Peripheral Venous Catheter‐Related Bloodstream Infections: Impact on Incidence and Mortality.” Journal of Hospital Infection 100, no. 3: e178–e186. 10.1016/j.jhin.2018.06.010. 29928942

[jan70347-bib-0066] Savage, T. J. , A. D. Lynch , and S. E. Oddera . 2019. “Implementation of a Vascular Access Team to Reduce Central Line Usage and Prevent Central Line‐Associated Bloodstream Infections.” Journal of Infusion Nursing 42, no. 4: 193–196. 10.1097/NAN.0000000000000328. 31283661

[jan70347-bib-0067] Schults, J. A. , P. Calleja , E. Slaughter , et al. 2022. “Peripheral Intravenous Catheter Insertion and Use of Ultrasound in Patients With Difficult Intravenous Access: Australian Patient and Practitioner Perspectives to Inform Future Implementation Strategies.” PLoS One 17, no. 6: e0269788. 10.1371/journal.pone.0269788. 35749443 PMC9231778

[jan70347-bib-0068] Sebbane, M. , P. G. Claret , S. Lefebvre , et al. 2013. “Predicting Peripheral Venous Access Difficulty in the Emergency Department Using Body Mass Index and a Clinical Evaluation of Venous Accessibility.” Journal of Emergency Medicine 44, no. 2: 299–305. 10.1016/j.jemermed.2012.07.051. 22981661

[jan70347-bib-0069] Sneyers, B. , C. Nyssen , P. Bulpa , et al. 2025. “Appropriateness of Intravenous Fluid Prescriptions in Hospitalised Patients: A Point Prevalence Study.” International Journal of Clinical Pharmacy 47, no. 1: 136–145. 10.1007/s11096-024-01816-9. 39527169

[jan70347-bib-0070] Steere, L. , C. Ficara , M. Davis , and N. Moureau . 2019. “Reaching One Peripheral Intravenous Catheter (PIVC) Per Patient Visit With Lean Multimodal Strategy: The PIV5Rights Bundle.” Journal of the Association for Vascular Access 24, no. 3: 31–43. 10.2309/j.java.2019.003.004.

[jan70347-bib-0071] Sweeny, A. , A. Archer‐Jones , S. Watkins , L. Johnson , A. Gunter , and C. Rickard . 2022. “The Experience of Patients at High Risk of Difficult Peripheral Intravenous Cannulation: An Australian Prospective Observational Study.” Australasian Emergency Care 25, no. 2: 140–146. 10.1016/j.auec.2021.07.003. 34456181

[jan70347-bib-0072] Taşdelen, Y. , A. Topan , and Ö. Öztürk Şahin . 2024. “Paediatric Nurses' Experiences of Success and Failure in First‐Time Peripheral Intravenous Catheter Insertion: A Qualitative Study.” Journal of Pediatric Nursing 75: 57–63. 10.1016/j.pedn.2023.12.002. 38101312

[jan70347-bib-0073] Timsit, J. F. , O. Mimoz , B. Mourvillier , et al. 2012. “Randomized Controlled Trial of Chlorhexidine Dressing and Highly Adhesive Dressing for Preventing Catheter‐Related Infections in Critically Ill Adults.” American Journal of Respiratory and Critical Care Medicine 186, no. 12: 1272–1278. 10.1164/rccm.201206-1038OC. 23043083

[jan70347-bib-0074] Timsit, J. F. , A. Tabah , and O. Mimoz . 2022. “Update on Prevention of Intra‐Vascular Accesses Complications.” Intensive Care Medicine 48, no. 10: 1422–1425. 10.1007/s00134-022-06763-5. 35768729

[jan70347-bib-0075] Ullman, A. J. , M. L. Cooke , M. Mitchell , et al. 2015. “Dressings and Securement Devices for Central Venous Catheters (CVC).” Cochrane Database of Systematic Reviews 2015, no. 9: CD010367. 26358142 10.1002/14651858.CD010367.pub2 PMC6457749

[jan70347-bib-0076] van Loon, F. , L. van Hooff , H. de Boer , et al. 2019. “The Modified A‐DIVA Scale as a Predictive Tool for Prospective Identification of Adult Patients at Risk of a Difficult Intravenous Access: A Multicenter Validation Study.” JCM 8, no. 2: 144. 10.3390/jcm8020144. 30691137 PMC6406455

[jan70347-bib-0077] van Loon, F. H. , L. A. Puijn , W. H. van Aarle , A. T. Dierick‐van Daele , and A. R. Bouwman . 2018. “Pain Upon Inserting a Peripheral Intravenous Catheter: Size Does Not Matter.” Journal of Vascular Access 19, no. 3: 258–265. 10.1177/1129729817747531. 29772984

[jan70347-bib-0078] van Loon, F. H. J. , M. P. Buise , J. J. F. Claassen , A. T. M. Dierick‐van Daele , and A. R. A. Bouwman . 2018. “Comparison of Ultrasound Guidance With Palpation and Direct Visualisation for Peripheral Vein Cannulation in Adult Patients: A Systematic Review and Meta‐Analysis.” British Journal of Anaesthesia 121, no. 2: 358–366. 10.1016/j.bja.2018.04.047. 30032874

[jan70347-bib-0079] van Loon, F. H. J. , L. A. P. M. Puijn , S. Houterman , and A. R. A. Bouwman . 2016. “Development of the A‐DIVA Scale: A Clinical Predictive Scale to Identify Difficult Intravenous Access in Adult Patients Based on Clinical Observations.” Medicine 95, no. 16: e3428. 10.1097/MD.0000000000003428. 27100437 PMC4845841

[jan70347-bib-0080] Wallis, M. C. , M. McGrail , J. Webster , et al. 2014. “Risk Factors for Peripheral Intravenous Catheter Failure: A Multivariate Analysis of Data From a Randomized Controlled Trial.” Infection Control and Hospital Epidemiology 35, no. 1: 63–68. 10.1086/674398. 24334800

[jan70347-bib-0081] Whalen, M. , B. Maliszewski , and D. L. Baptiste . 2017. “Establishing a Dedicated Difficult Vascular Access Team in the Emergency Department: A Needs Assessment.” Journal of Infusion Nursing 40, no. 3: 149–154. 10.1097/NAN.0000000000000218. 28419011

[jan70347-bib-0082] Witting, M. D. 2012. “IV Access Difficulty: Incidence and Delays in an Urban Emergency Department.” Journal of Emergency Medicine 42, no. 4: 483–487. 10.1016/j.jemermed.2011.07.030. 22137793

[jan70347-bib-0083] World Health Organization , ed. 2024. Guidelines for the Prevention of Bloodstream Infections and Other Infections Associated With the Use of Intravascular Catheters: Part 1: Peripheral Catheters. World Health Organization. 38810002

[jan70347-bib-0084] Xu, H. G. , A. J. Ullman , C. M. Rickard , and A. Johnston . 2023. “Factors Impacting Emergency Department Clinicians' Peripheral Intravenous Catheter Practice: A Qualitative Analysis.” International Emergency Nursing 71: 101366. 10.1016/j.ienj.2023.101366. 37852059

[jan70347-bib-0085] Yalçınlı, S. , F. K. Akarca , Ö. Can , A. Şener , and C. Akbinar . 2019. “Factors Affecting the First‐Attempt Success Rate of Intravenous Cannulation in Older People.” Journal of Clinical Nursing 28, no. 11–12: 2206–2213. 10.1111/jocn.14816. 30786094

[jan70347-bib-0086] Zingg, W. , A. Barton , J. Bitmead , et al. 2023. “Best Practice in the Use of Peripheral Venous Catheters: A Scoping Review and Expert Consensus.” Infection Prevention in Practice 5, no. 2: 100271. 10.1016/j.infpip.2023.100271. 36910422 PMC9995289

