# Supplementary material for: Procedural and Therapy Peripheral Intravenous Catheters: A Framework for Safety, Quality and Patient‐Centred Care
Source: J Adv Nurs. 2025 Nov 3;82(8):7748–60. doi: 10.1111/jan.70347 (PMC13356444; doi:10.1111/jan.70347)
Supplement: Supplementary file 1 — Data S1: Research strategy. [file JAN-82-7748-s001.docx]

Supplementary file 1. Research strategy.

**Databases**

PubMed

**Date searched**

November 25th 2024

**PubMed** 1304 results

Includes MeSH

**(**(("peripheral"[tiab] OR "peripherally"[tiab]) AND ("Administration, Intravenous"[Mesh] OR "Veins"[Mesh] OR "venous"[tiab] OR "intravenous"[tiab] OR “IV”[tiab] OR "vascular"[tiab] OR "intravascular"[tiab] OR “vein”[tiab] OR “veins”[tiab]) AND ("Catheters"[Mesh] OR "Catheterization"[Mesh] OR "catheter"[tiab] OR "catheters"[tiab] OR "catheterization"[tiab] OR "catheterisation"[tiab] OR "cannulation"[tiab] OR "cannula"[tiab] OR "cannulas"[tiab] OR "cannulae"[tiab])) OR ("Catheterization, Peripheral"[Mesh] OR "PVC"[tiab] OR "PVCs"[tiab] OR "PIVC"[tiab] OR "PIVCs"[tiab] OR “PIV”[tiab] OR “PIVs”[tiab])**) AND (**"Guidelines as Topic"[Mesh] OR "Guideline" [Publication Type] OR "Consensus"[Mesh] OR "Consensus Development Conferences as Topic"[Mesh] OR "Consensus Development Conference" [Publication Type] OR “guideline”[tiab] OR “guidelines”[tiab] OR “best practice”[ti] OR “consensus”[ti] OR “recommendation”[ti] OR “recommendations”[ti] OR “policy”[ti] OR “policies”[ti] OR “standard”[ti] OR “standards”[ti] OR “evidence-based”[ti] OR “evidence”[ti]**) AND**1990:2024[dp] **AND** (eng[la] OR und[la]) **NOT**(animals [mh] NOT humans [mh])

Eligible publications focused on PIVC insertion, maintenance, or removal, especially regarding dwell time and complication management. Included sources were clinical guidelines, expert consensus statements, institutional protocols, and narrative reviews, limited to publications in English or French. Studies exclusively on central venous catheters, unrelated to PIVC management, or lacking methodological detail were excluded.

Findings were narratively synthesised to highlight key themes in PIVC management, particularly how guidelines and practices differentiate between short- and long-term use, manage complications, and allocate resources. This analysis identified evidence gaps and areas for future research.
